# The PD-1/PD-L1 Checkpoint in Normal Germinal Centers and Diffuse Large B-Cell Lymphomas

**DOI:** 10.3390/cancers13184683

**Published:** 2021-09-18

**Authors:** Marcos Garcia-Lacarte, Sara C. Grijalba, Javier Melchor, Adrián Arnaiz-Leché, Sergio Roa

**Affiliations:** 1 Department of Biochemistry and Genetics, University of Navarra, 31008 Pamplona, Spain; mglacarte@unav.es (M.G.-L.); scontreras.3@alumni.unav.es (S.C.G.); jmelchor@alumni.unav.es (J.M.); aarnaiz.3@alumni.unav.es (A.A.-L.); 2 Hemato-Oncology Program, Cima University of Navarra, 31008 Pamplona, Spain; 3 Navarra Institute for Health Research (IdiSNA), 31008 Pamplona, Spain; 4 Network Center for Biomedical Research in Cancer—Centro de Investigación Biomédica en Red de Cáncer (CIBERONC), Institute of Health Carlos III, 28029 Madrid, Spain

**Keywords:** immune checkpoint, germinal center, lymphoma, GC B cells, T follicular helper cells, T follicular regulatory cells, B regulatory cells, combination immunotherapy

## Abstract

**Simple Summary:**

The PD-1/PD-L1 axis is not only involved in anti-tumour immune evasion of germinal center (GC)-derived diffuse large B cell lymphomas (DLBCL), but also inherently in the fine-tuned regulation of normal GC reactions during humoral immune responses. This checkpoint axis modulates crosstalks between B and T cells that allow positive selection for survival and proliferation. Malignant DLBCL cells may deceive and take advantage of these mechanisms to establish an immunosuppressive microenvironment. This review delves into PD-1/PD-L1 role in the complex inter-cellular interactions from normal GC reactions to DLBCL progression, in order to highlight vulnerabilities that could be targeted by promising combination immunotherapies.

**Abstract:**

Besides a recognized role of PD-1/PD-L1 checkpoint in anti-tumour immune evasion, there is accumulating evidence that PD-1/PD-L1 interactions between B and T cells also play an important role in normal germinal center (GC) reactions. Even when smaller in number, T follicular helper cells (T_FH_) and regulatory T (T_FR_) or B (Breg) cells are involved in positive selection of GC B cells and may result critical in the lymphoma microenvironment. Here, we discuss a role of PD-1/PD-L1 during tumour evolution in diffuse large B cell lymphoma (DLBCL), a paradigm of GC-derived lymphomagenesis. We depict a progression model, in two phases, where malignant B cells take advantage of positive selection signals derived from correct antigen-presentation and PD-1/PD-L1 inter-cellular crosstalks to survive and initiate tumour expansion. Later, a constant pressure for the accumulation of genetic/epigenetic alterations facilitates that DLBCL cells exhibit higher PD-L1 levels and capacity to secrete IL-10, resembling Breg-like features. As a result, a complex immunosuppressive microenvironment is established where DLBCL cells sustain proliferation and survival by impairing regulatory control of T_FR_ cells and limiting IL-21-mediated anti-tumour functions of T_FH_ cells and maximize the use of PD-1/PD-L1 signaling to escape from CD8^+^ cytotoxic activity. Integration of these molecular and cellular addictions into a framework may contribute to the better understanding of the lymphoma microenvironment and contribute to the rationale for novel PD-1/PD-L1-based combinational immunotherapies in DLBCL.

## 1. Introduction

Interest in the immune-checkpoint protein programmed death 1 (PD-1), in T lymphocytes, and its ligand (PD-L1), in lymphoma B cells, have increased in parallel to the remarkable clinical outcomes demonstrated with their blockade in a broad range of tumour types [1]. Beyond its role in anti-tumour immune evasion [1,2], this PD-1/PD-L1 pathway is also inherently necessary to maintain peripheral tolerance and attenuate potentially dysregulated or damaging T-cell responses [3,4]. This is especially relevant within germinal centers (GCs) at secondary lymphoid organs, where the correct orchestration of B and T cell interactions is critical for B cell activation and efficient humoral responses [5,6]. Indeed, failure of appropriate T cell signals during GC reactions results in impaired GC maintenance and immune response [7,8] and may contribute to other genetic and epigenetic determinants in GC-derived lymphomagenesis [9,10]. Particularly, diffuse large B cell lymphoma (DLBCL) is the most common lymphoid malignancy in adults worldwide and has long been regarded as a paradigm of aggressive disease originated from GC-experienced B cells [11,12]. In this review, we describe major evidence for naturally occurring PD-1/PD-L1 signaling to fine modulate GC reactions and discuss evidences for how GC-derived malignant cells may exploit this immune-checkpoint to facilitate selection and survival first, and elude anti-tumour immune responses later at advanced DLBCL stages. With the development of the immune-oncology field and the advent of promising novel immunotherapy treatments, integration of these vulnerabilities into a framework may contribute to the rationale for PD-1/PD-L1-related combinational immunotherapy in DLBCL.

## 2. The PD-1/PD-L1 Axis during the Germinal Center Reaction

**Figure 1:**
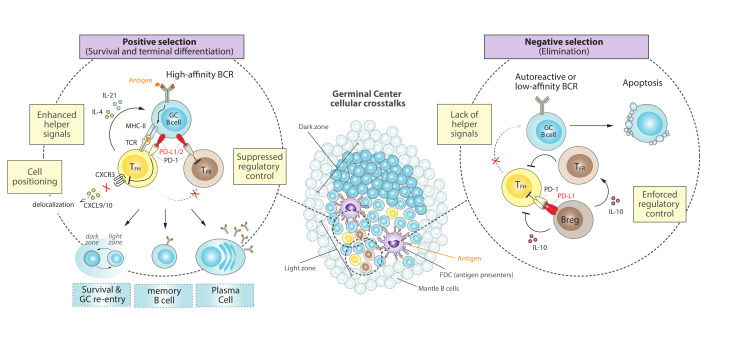
Cellular crosstalks and the PD-1/PD-L1 axis during normal GC reactions. Surrounded by a mantle zone, GCs expand as a result of the fast proliferation of antigen-specific B cells that are densely packed to form a dark zone, before moving into a light zone containing FDCs and other regulatory cells that orchestrate selection. (**Left**) B cells that express high-affinity BCR lead to greater antigen capture and higher density of peptide-MHC-II presentation, engaging PD-1/PD-L1 signaling that promotes T_FH_ cell positioning and signaling, suppression of regulatory control and positive selection of B cells. Survival and recirculation between the dark and light zones facilitate several iterative rounds of BCR mutation and selection, which ultimately leads to terminal differentiation into memory or antibody-secreting plasma cells. (**Right**) A tight regulation is necessary to avoid autoreactive or low-affinity B cells, which are unable to capture sufficient antigen and undergo apoptosis. This is also enforced by PD-L1^hi^ Bregs and T_FR_ cells, which dampen T_FH_ signals and further lean towards negative selection of undesired B cells. FDC, follicular dendritic cells; Breg, regulatory B cell; T_FH_; T follicular helper; T_FR_, T follicular regulatory; BCR, B-cell receptor; TCR, T-cell receptor; MHC-II, major histocompatibility complex-II; CXCR3, C-X-C chemokine receptor type 3; CXCL9/10, C-X-C motif chemokines 9 or 10.

The continuous interactions of B cells with the small fraction of various T cell populations before and during GC reactions, have been shown to be a critical limiting factor for GC maintenance and selection [6,7,8]. Only those B cells with higher affinity to the antigen are selected and clonally expanded, whereas lower affinity B cells undergo apoptosis and are dismissed [6,10,13]. Beyond the essential signaling through the B cell receptor (BCR) and the amount of antigen peptide on major histocompatibility complex-II (pMHC-II) displayed on the cell surface [14,15], T cell-derived signals work complementarily to ensure the efficiency of GC selection and preventing autoimmunity or GC lymphomagenesis. We review here that, in addition to the well-known costimulatory CD40/CD40L axis [16], CXCR4, ICOS or T-cell secreted cytokines such as IL-4 or IL-21 [17,18,19], there are accumulating evidences that immune-checkpoint signals through PD-1/PD-L1 interactions between B and T cells play an important role in GC reaction (Figure 1).

### 2.1. PD-1 in T Follicular Helper (T_FH_) Cells

The microanatomical distribution of PD-1 in human tonsils demonstrated that it was expressed on most T cells in the light zone (LZ) of GCs [20], which is the site where GC B cells diversified by hypermutation are selected [6]. This LZ is rich in follicular dendritic cells (FDCs) and in a smaller population of T follicular helper (T_FH_) cells that are critical for positive selection of B cells [13] (Figure 1, center). Indeed, a specific population of CD4^+^ T_FH_ cells, which are also CXCR5^+^, ICOS^+^ and PD1^+^, have demonstrated to be fundamental during GC formation and a crucial source of cytokines required for a correct GC expansion and B cell isotype class switching [8,21,22]. The high expression of PD-1 by T_FH_ cells inside the GC territory [23] might simply reflect experience of antigen stimulation [24]. However, PD-1 engagement in T_FH_ cells can attenuate ICOS signaling [25], which is critical for T_FH_ generation [22], and suppresses overall T_FH_ cell development [24,26]. This is consistent with the observations in PD ligands- or PD-1-deficient mice that the lack of PD-1 signaling results in more CD4^+^ T_FH_ cells [27,28]. However, these PD-1 signaling-deficient T_FH_ cells failed to promote GC B cell survival, either because they showed a diminished capacity to synthesize the important pro-survival cytokines IL-4 and IL-21 (in the absence of PD-1) [27,29] or, on the contrary, because they displayed a hyperactive phenotype with increased IL-21 production and expression of FasL that promotes GC B cell death (in the absence of PD-L1 alone) [28]. These apparently contradictory T_FH_ phenotypes might be capturing differences in PD-1 signaling by PD-L1 or PD-L2, but ultimately reinforce the idea that despite the negative role of PD-1 in T_FH_ development, PD-1 is required for optimal IL-21 production by T_FH_ cells and tight regulation of GC B cell survival [24,27,30] (Figure 1, left).

Another positive impact of PD-1 function in GC biology occurs during the orchestration of the dynamic follicular environment, where PD-1 initially ensures that activated T_FH_ cells that are recruited into follicles are those that highly co-express ICOS and PD-1 [24,31]. Then, PD-1 helps to concentrate T_FH_ cells in GCs by restricting CXCR3 expression and therefore preventing delocalization to interfollicular regions in response to CXCL9 and/or CXCL10 [24]. Furthermore, PD-1 can dampen T cell receptor (TCR) ligand sensitivity [32,33] and thereby help to promote affinity-based selection by T_FH_ cells through enforcing a more stringent selection threshold for competing GC B cells [24]. Altogether, these studies support the idea that signaling through PD-1 by CD4^+^ T_FH_ is essential to control both proper positioning and helper functions to GC responses (Figure 1, left).

On the other hand, CD8^+^ T cells have been traditionally considered as effector cytotoxic cells that protect against viruses and tumour cells releasing cytokines or differentiating into cytotoxic T lymphocytes [34,35]. However, distinct novel CXCR5^+^ PD-1^+^ subset of CD8^+^ T cells have recently been identified, which can directly help B cells to produce antibodies in vitro and shares similar gene expression signatures with CD4^+^ T_FH_ cells, including the production of IL-21 [36]. Additional studies demonstrate the existence of CXCR5^+^ CD8^+^ that can differentiate into T_FH_ cells [37,38], and may also express ICOS and secrete IL-21 [39,40,41], further helping B cells to form GC, proliferate and produce antibodies.

### 2.2. PD-1 in T Follicular Regulatory (T_FR_) Cells

GCs reactions are not only controlled by helper signals that promote selection and proliferation, but also by follicular regulatory T cells (T_FR_) that suppress the magnitude and output of the GC response [42,43] (Figure 1, right). These T_FR_ cells are a subset of CD4^+^ FOXP3^+^ regulatory T (Treg) cells that are located within the GC and, like T_FH_ cells, express CXCR5 (although CXCR5-independent T_FR_ mechanisms might also exist) [44], ICOS and PD-1 [43,45,46]. Although the precise mechanisms by which T_FR_ cells control GC B cell responses are still not fully understood, it is thought that T_FR_ cells impair GC reactions by suppressing T_FH_ proliferation and by inhibiting the production of T_FH_ derived cytokines that are crucial for B cell selection and survival [47,48]. Depletion of T_FR_ results in increased numbers of T_FH_ and GC B cells [45], reinforcing the key role of T_FR_ in suppressing GC reactions through the control of T_FH_ cells.

PD-1 is also expressed in T_FR_, and a lack of PD-1 or its ligand PD-L1 results in a dramatic increase of T_FR_ numbers in the draining lymph node of immunized mice, which is accompanied by a hyperactive suppressive capacity [49]. Therefore, PD-1 signaling may inhibit the number of T_FR_ cells, even if the abundance of T_FH_ cells is not immediately affected, skewing the T_FR_/T_FH_ ratio towards a more helper phenotype that promote B cell survival and selection (Figure 1, left).

Although the interest in regulatory T cells has focused principally in CD4^+^ FOXP3^+^ cells, there is increasing attention to a subpopulation of CD8^+^ that may regulate GC responses and suppress T_FH_ action [50]. Interestingly, in humans the majority of follicular CD8^+^ T cells are T_FR_ that can induce T_FH_ apoptosis and inhibit IL-21 production [51]. PD-1 has been described to be expressed in CD8^+^ T_FR_ cells and to be necessary for their correct suppressive function [52], further supporting a fine-tuned involvement of PD-1/PD-L1 signaling in T cell regulatory functions.

### 2.3. PD-L1 Expression and PD-1 Downregulation in GC B Cells

PD-L1 is widely expressed by many different immune cells, while PD-L2 expression is much more restricted. Both of them, however, can be expressed on GC B cells [27,28,49], promoting PD-1 signaling in T_FH_ and T_FR_ cells, as discussed above, and thereby influencing humoral immune responses (Figure 1, left). In fact, PD-L1, but not PD-L2, can already be found on splenic resting B cells and may be up-regulated by stimulation with anti-IgM or LPS [53]. Although the underlying mechanisms for the regulation of PD-L1 expression in GC B cells remain poorly understood, the master GC regulator BCL6 has been shown to act as a key transcriptional repressor of PD-L1 and PD-L2 to dampen excessive PD-1 signaling on both T_FH_ and T_FR_ cells [10,54,55]. Consistently, BCL6-expressing mature GC B cells initially express low levels of PD-1 ligands [54] while late GC B cells, which turn down BCL6 to initiate terminal differentiation, upregulate PD-1 ligands and seem to be more sensitive to PD-L1/2-PD-1 signaling [27].

On the other hand, PD-1 can co-cluster with the BCR and inhibit B-cell activation and proliferation [56,57], suggesting analogous co-inhibitory receptor functions for PD-1 in both T and B cells. Consistent with this, PD-1 expression appears specifically downregulated on GC B cells and re-acquired upon GC exit, supporting a role of PD-1 in B cells as a guardian of uncontrolled activation [56].

In summary, these observations support the notion that PD-1 turnoff and PD-L1 expression in late GC B cells conditionate their selection and survival, where the interaction between PD-1-ligands on B cells and PD-1 on T cells is required for the optimal output of the GC response.

### 2.4. PD-L1 and PD-1 Expression in Regulatory B (Breg) Cells

There is accumulating evidence for the existence of a subset of B cells that can exert immune regulatory functions, termed regulatory B cells (Breg) [58,59,60,61]. The majority of studies on the inhibitory function of Bregs have focused on IL-10, IL-35 and TGF-β as key players that can repress the differentiation, proliferation and effector function of various cell types, like dendritic, CD4^+^ and CD8^+^ T cells [62,63,64,65,66]. A role for Bregs during GC reaction responds to its ability to restrict T_FH_ maturation and expand T_FR_ cells, inhibiting subsequent antibody responses [62,67] (Figure 1, right).

Independently of IL-10 production, other suppressive mechanisms have been reported whereby Bregs can dampen T cell responses [63,68]. For example, Breg cells can induce CD4^+^ T cell death by expressing FasL [69], and can dramatically suppress humoral responses through the expression of high levels of PD-L1 (PD-L1^hi^ Breg cells) [67]. This is due to PD-L1^hi^-mediated restriction of the expansion and function of T_FH_ cells, partially caused by the activation of the negative regulator of T_FH_ differentiation STAT5 [70,71]. This mechanism resulted independent from IL-10 and Treg cells, and susceptible to PD-L1 blockade, indicating a direct connection between Breg and T_FH_ cells through the PD-1/PD-L1 axis [67], highlighting how PD-L1^hi^ Bregs might ultimately impact GC B cell responses (Figure 1, right).

Interestingly, Breg cells are not restricted to a determined B sublineage but can be instead remodeled from B cells at different stages of development in response to different stimuli such as infections, inflammatory processes or other malignancies [72,73]. One example are regulatory plasmocytes, B cells in terminal differentiation towards antibody secretor plasma cells that in the presence of some antigens can be transcriptionally reprogramed to produce high levels of inhibitory molecules as IL-10, PD-L1 and LAG-3, repressing both myeloid and lymphoid pro-inflammatory populations [74,75]. Other examples are protumorigenic Breg populations that can affect anti-tumour immune responses through different suppressive mechanisms [61,72]. Certain subsets of these tumour-infiltrating Breg cells can upregulate PD-1 expression in response to signaling from cancer cells, promoting T-cell dysfunction via IL-10 [76] or, independently of IL-10, via PD-L1 [77]. In these cases, immune-checkpoint blockade demonstrated that PD-1-triggered production of IL-10 and PD-L1-mediated immunosuppression of T cells were instrumental for Breg protumorigenic functions. Therefore, the PD-1/PD-L1 axis may be utilized by regulatory B cells in different ways, and its blockade might offer a multifaceted benefit that recommend further investigation.

## 3. The PD-1/PD-L1 Axis in DLBCL

**Figure 2:**
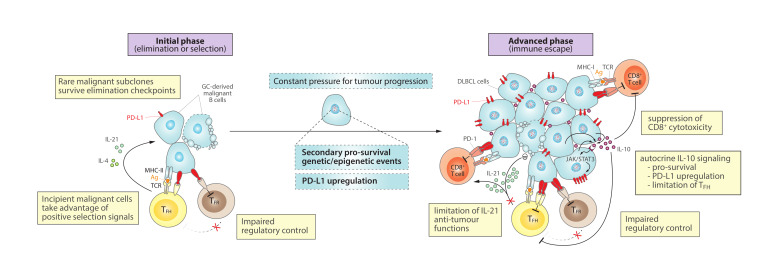
Role of the PD-1/PD-L1 axis and cytokine dysregulation in a progression model for DLBCL. (**Left**) At an initial phase, incipient malignant B cells that arise during GC reactions might take advantage of positive selection signals derived from correct antigen-presentation and PD-1/PD-L1 checkpoint in order to survive and initiate clonal expansion. Then, constant pressures during tumour evolution promote the acquisition of additional genetic/epigenetic mutations and PD-L1 upregulation, contributing to tumour maintenance and progression. (**Right**) At more advanced stages, DLBCL cells tend to exhibit higher PD-L1 levels and capacity to secrete IL-10, which cooperatively promote an immunosuppressive microenvironment that helps DLBCL to escape from anti-tumour CD8^+^ cytotoxic activity and sustain proliferation and survival, impairing regulatory control by T_FR_ cells and limiting IL-21-mediated anti-tumour functions of T_FH_ cells. MHC-I, major histocompatibility complex-I; Ag, antigen.

The aforementioned proper regulation of GC cells is crucial for a correct immune response, since dysregulation of proliferation, mutation and differentiation of B cells undergoing GC reactions may lead to lymphomagenesis and DLBCL. Indeed, there is an increasing appreciation of the role of the composition, function and localization of immune and stromal determinants of the tumour microenvironment in DLBCL [78,79,80,81,82,83]. Here, we review current evidence for the implication of the PD-1/PD-L1 axis in DLBCL and propose a progression model, in two phases, where increased PD-1/PD-L1 signaling and dysregulated cytokines promote anti-tumour immune escape (Figure 2). Integration of all these molecular vulnerabilities will hopefully help expanding our knowledge on the role of the microenvironment in DLBCL pathobiology and contribute to the rationale for novel PD-1/PD-L1-related combination immunotherapies for this disease.

### 3.1. Initial Phase: Elimination or Selection of GC-Derived Malignant Cells

During the initial state of malignant transformation from GC B cells to DLBCL cells, it is plausible that PD-1/PD-L1-related mechanisms governing normal GC reactions, as described above (Figure 1), may also be deceived into driving clonal selection and DLBCL evolution. In this early scenario, PD-L1^+^ transformed GC cells might continue suppressing regulatory control by T_FR_ cells and skew the T_FH_/T_FR_ ratio towards a helper phenotype, taking advantage of positive signals to enhance survival and selection of malignant subclones (Figure 2, left). Indeed, the study of DLBCL patient samples showed an enrichment of the T_FH_ subset compared to healthy controls [84]. Then, following the general principles of cancer evolution [85], a continuous pressure for additional genetic and epigenetic alterations may provide growth advantage to selected malignant subclones, permitting DLBCL expansion.

#### 3.1.1. Secondary Pro-Survival and Genetic/Epigenetic Events during DLBCL Progression

Numerous studies have demonstrated the large amount of genetic and epigenetic heterogeneity that characterize DLBCL [9,86,87]. How all these molecular events interact as driver or passenger alterations to impact GC B-cell fate is not yet fully understood, but they do reveal the existence of distinct DLBCL subgroups with unique biological properties and clinical behaviors that reflect cell-of-origin transcriptional hallmarks [11,88], co-occurrence of genetic alterations [89,90,91] or different microenvironment compositions [79,81]. These DLBCL hallmarks have multifaceted roles promoting cancer progression, including at times (see next section) the regulation and promotion of PD-L1 expression itself.

#### 3.1.2. PD-L1 Upregulation in DLBCL

Upregulation of PD-L1 has been previously associated with rapid progression and poor prognosis of DLBCL [92,93,94]. Genetic abnormalities at the chromosome 9p24.1 represent crucial mechanisms affecting PD-L1 expression, but less than 27% of DLBCL patients carry copy number gains, amplifications, or translocations affecting the PD-L1/L2 locus [95,96,97,98]. In addition, only 8% of DLBCL cases exhibit structural variations disrupting the 3′-UTR of the PD-L1 gene that lead to the stabilization and elevation of aberrant PD-L1 transcripts [99]. Yet PD-L1 expression is highly diverse in DLBCL, ranging from 25–70% [100], which might reflect differences in the dynamics of tumour progression and associate more clearly with selected lymphoma subtypes. Epigenetic modulators, including promoter DNA methylation or histone modifications [101,102], might also be at the basis for the upregulation of PD-L1 without underlying genetic alterations at 9p24.1. Such heterogeneity in the prevalence of PD-L1 expression might be the reason for the current disappointing low rate of clinical responses to PD-1/PD-L1 immune-checkpoints in DLBCL patients [103,104], as well as for the controversial prognostic significance of PD-L1 expression with studies reporting its association with poorer outcome [92,93,94,96,105,106], undetectable prognostic significance [107], or trends towards better event-free survival [97]. Interestingly, PD-L1 expression is more frequently observed in the prognostically unfavorable non-GCB/activated B cell-like (ABC) subtype [92,93,94,95,105,107,108], in non-transformed de novo cases [108], or in EBV-positive DLBCL cases [107]. Indeed, PD-L1 levels escalate after infection with different viruses related with poorer prognosis of DLBCL, including Epstein–Barr virus (EBV) [109], hepatitis B virus (HBV) [110], and human immunodeficiency virus (HIV) [111].

During tumour evolution, high levels of MYC expression can result in increased expression of PD-L1 by direct binding to its promoter [112], as well as by hijacking the phospho-eIF2a dependent adaptive stress pathway to bypass the post-transcriptional control orchestrated at the 5′-UTR of the PD-L1 mRNA [113]. On the other hand, inactivating mutations of TP53, which can be detected in approximately 20% of DLBCL patients [89,114,115,116], have been shown to increase PD-L1 expression in various tumour types [101,117,118] and might result in defective p53-regulated miRNA responses that normally control PD-L1 levels [119,120]. Indeed, noncoding RNAs that may target PD-L1, such as miR-34a [120,121] or miR-195 [122] in DLBCL, as well as other immune-checkpoints, are receiving increased attention [123,124]. Hypoxia, an inevitable hallmark of tumour microenvironment resulting from deprivation of oxygen due to the abnormal vasculature and tumour mass expansion, may also enforce gene expression reprogramming in DLBCL [125] and can induce PD-L1 expression [126].

The constitutive activation of NF-kB and JAK/STAT3 signaling is also frequently associated with the most aggressive nonGCB/ABC-DLBCL subtype [127], and a coordinated activity of both pathways has been demonstrated to be important in the metabolic reprogramming of DLBCL cells [128]. NF-kB and JAK/STAT3 activation can be driven either by a complex microenvironment or intrinsic genetic lesions and are emerging as key positive regulators of PD-L1 expression [129,130,131,132]. Consistent with this idea, we recently described that NF-kB-driven lymphomagenesis in mouse models of ABC-DLBCL is strongly associated to PD-L1 upregulation and immune evasion [133], which can be efficiently blocked to improve long-term control of lymphomas and support the potential of the PD-1/PD-L1 axis as an attractive target for lymphoma immunotherapy.

### 3.2. Advanced Phase: Immune Escape by DLBCL Cells

As a result of the accumulation and selection of the above discussed traits, a more advanced scenario could be described where DLBCL cells have consolidated their ability for immune evasion, taking advantage of (i) preserved “select me” and “do not kill me” signals inherited from the GC B cell-of-origin, and (ii) extended mechanisms that further contribute to impaired T cell infiltration and/or cytotoxic functions (Figure 2, right). In support of this notion, deconvolution of transcriptional signatures from DLBCL microenvironment cells suggested that disease progression tends to associate with immune desertion and decreasing T cell infiltration, resulting in poorest clinical outcomes [81]. Activated T helper cells, as well as other associated regulatory T or B cells, normally release cytokines (e.g., IL-10, IL-21) into the immunological synapse toward the antigen-presenting GC B cells, which may result in dysregulation within the DLBCL microenvironment in parallel to alterations in tumour-infiltrating T cell populations. While some of these cytokines are essential for GC homeostasis (Figure 1), they may become detrimental by exerting immunosuppressive and pro-survival activities that ultimately facilitate tumour progression (Figure 2). Next, we review how DLBCL cells at more advanced stages can mimic some features of Breg cells, including high PD-L1 expression and IL-10 secretion, and promote an immunosuppressive microenvironment that reinforce anti-tumour immune evasion.

#### 3.2.1. Autocrine IL-10 Signaling

Increasing evidences demonstrate that B cells can also produce IL-10 to mediate regulatory functions [73,134], which can even stimulate B cell proliferation under certain local conditions [135,136]. In DLBCL, increased levels of IL-10 frequently associate with the ABC-DLBCL subtype [137], and in-vitro experiments have demonstrated that DLBCL cells can sustain proliferation and survival by autocrine IL-10/JAK/STAT3 signaling [137,138]. Beyond this pro-survival effect, IL-10 production by the DLBCL cells could also promote immunosuppression by PD-L1 upregulation through intracellular mechanisms that involve JAK/STAT3 signaling [132], and mimic Breg-derived IL-10 secretion to suppress CD8 cytotoxic responses [61,73] (Figure 2, right). Yet, the exact role of IL-10 in B cell lymphomas remains controversial and urges further investigation, especially since IL-10 receptor (IL-10R) deficiency associates with lymphoma predisposition [139] and IL-10 may also possess anti-tumour functions by promoting CD8^+^ T cell responses in certain scenarios, including DLBCL [140,141,142]. Furthermore, IL-10 can also disturb the ratio of T_FH_/T_FR_ cell populations (see below), which might exert a profound impact on lymphoma progression.

#### 3.2.2. Impaired T-Cell Regulatory Control

The role of regulatory T cells is more intricate in hematological cancers compared to other tumour types. On the one hand, the number of tumour infiltrating T_FR_ cells is frequently higher in DLBCL patients with less severe clinical presentation of DLBCL, but reduced in advanced DLBCL stages [143]. Similarly, a positive association between the number of intra-tumour FOXP3^+^ Treg cells and a better prognosis of DLBCL has been observed [144,145,146]. In fact, primary human co-cultures with Treg cells can induce the apoptosis of tumour B cells [147], and transformation of follicular lymphoma to DLBCL associates with decreased numbers of intra-tumour Tregs [148]. Altogether, these observations support a general protective role of intra-tumour Tregs in trying to restrict T_FH_-mediated initial pro-tumour support or as a surrogate marker for active anti-tumour immune responses [149]. PD-L1 upregulation may become an effective way to suppress any potential benefit from Treg cells in DLBCL, and help explaining the relative decrease in Treg numbers observed in murine PD-L1^+^ DLBCL [133].

On the other hand, Tregs might support B-cell tumour growth by efficiently inhibiting anti-tumour responses [149] and by locally secreting IL-10 with pro-survival activity for the malignant B cells [136]. In this sense, increased levels of Tregs are generally found in blood [143,150] and tumour [143] of DLBCL patients compared to healthy controls, and some studies have associated increased number of these Tregs with adverse clinical outcome for DLBCL [150,151], but not always [152]. Therefore, while it remains unclear the ultimate beneficial or detrimental contribution of Tregs to the DLBCL microenvironment, future studies should also take into consideration that there are various subsets of FOXP3^+^ Tregs with multifaceted functions [153,154]. In this regard, T_FR_ is a specialized subset of Tregs with particular roles in the control of T_FH_-driven GC responses [42,43,155], likely prone towards suppressive functions against the GC-derived lymphoma cells. Following this notion, DLBCL cells might successfully counteract T_FR_ cell expansion and its possible protective functions, by upregulating PD-L1 at advanced stages of the disease (Figure 2, right). Even in an alternative scenario with increased T_FR_ numbers, DLBCL cells might also be able to take advantage of IL-10 locally produced by these cells, demonstrating a complex role of T_FR_ cells within the tumour microenvironment of DLBCL.

#### 3.2.3. Limitation of T_FH_ Expansion and IL-21 Anti-Tumour Functions

It has been noted that IL-21 exerts diverse regulatory effects on healthy and tumour cells depending on the type of cell, stage of differentiation and type of stimulus. After proper BCR signaling and T cell-dependent B cell response, IL-21 can induce B cell proliferation and sustain normal GC reactions [156,157] (Figure 1, left). These IL-21 pro-survival properties could also play a role during initial stages of GC-derived malignant transformation (Figure 2, left), and even constitutively persist in some hematological malignancies (i.e., follicular lymphoma, multiple myeloma and Hodgkin’s lymphoma) [158]. However, opposite outcomes are possible in more advanced stages of DLBCL, where T_FH_-derived IL-21 might elicit anti-lymphoma activities through at least two different mechanisms (Figure 2, right). On one hand, IL-21 has demonstrated anti-DLBCL activity through the activation of STAT3-c-Myc signaling pathway and downregulation of the anti-apoptotic Bcl-2/Bcl-XL genes [157,159]. Indeed, cytotoxic activities of IL-21 have been observed for inappropriately activated B cells and some lymphoma cells, by induction of apoptosis and growth arrest [156,159,160,161,162]. On another hand, IL-21 might also become toxic for lymphomas by expanding and enhancing tumour-infiltrating cytotoxic CD8^+^ cells and NK cells [163,164,165,166]. Such immunotherapeutic potential of IL-21 to stimulate anti-tumour immune responses is being clinically investigated in different cancers [160,167,168].

A priori paradoxical, the number of circulating CD4^+^ T_FH_ cells, which likely reflects the type of T_FH_ cells infiltrating tumours, appears to be significantly increased in DLBCL cases, especially in more advanced stages of the disease [84]. However, these T_FH_ cells secrete significantly less IL-21 than other circulating CD4^+^ T cells [169] and associate with decreased serum IL-21 levels [170], suggesting a severe limitation of the expected anti-tumour functions of T_FH_ cells in DLBCL (Figure 2, right). This would be consistent with an immunosuppressive tumour microenvironment where DLBCL cells may directly restrict T_FH_ functions through increased surface PD-L1 expression and autocrine IL-10 secretion, acquiring immune evasion characteristics that resemble Breg-like functions [58,59,61,63,67,72,73]. These IL-21-impaired T_FH_ cells in DLBCL may also promote a cytokine switch towards IL-10 production by T_FR_ cells [169,171], further contributing to lymphoma maintenance and progression.

#### 3.2.4. Suppression of CD8 Cytotoxicity

It is plausible to expect that tumour-infiltrating CD8^+^ effector T cells may result immunosuppressed in DLBCL, as a consequence of aforementioned mechanisms of cytokine dysregulation (decreased T_FH_-derived cytotoxic IL-21 and increased autocrine IL-10) and PD-L1 upregulation, compromising efficient anti-lymphoma immune responses and facilitating immune escape (Figure 2, right). Consistent with this, the amount of intra-tumour PD-1^+^ T cells correlates positively with PD-L1^+^ lymphoma B cells in human [100,107,172] and murine DLBCL [133]. Interestingly, a high number of PD-1^+^ tumour-infiltrating lymphocytes is a favorable prognostic factor in DLBCL [107,173], suggesting ongoing anti-lymphoma T cell responses, still with a chance to respond to combinational PD-1/PD-L1 blockade [133] and yet not fully progressed into the immune deserted scenario with poorer clinical outcomes [81]. Altogether, these observations suggest that, while still controversial due to technical differences, challenges and cut-off values in PD-1 detection [100,174], there is a biological connection between CD8^+^ PD-1^+^ effector T cells and DLBCL progression with potential prognostic and clinical relevance that justifies further research for this fatal disease.

## 4. Immunotherapy Perspectives in DLBCL

**Figure 3:**
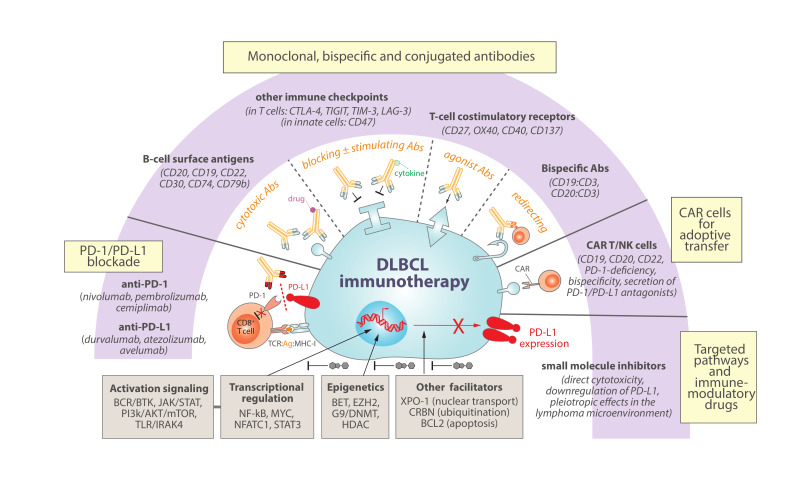
Immunotherapy strategies for DLBCL with promising rationale to involve or cooperate with PD-1/PD-L1 blockade. Combinational immunotherapy offers a potential to improve the current limited therapeutic efficacy of PD-1/PD-L1 blockade in DLBCL. For example, exploring novel monoclonal, bispecific or conjugated antibodies might cooperate to target a continuously extending portfolio of B-cell specific and tumour antigens, to block other immune-checkpoint inhibitors, to enhance immune stimulation by cytokine delivery or T-cell costimulatory receptor targeting, or to strategically redirect T cells trying to enhance anti-tumour engagements. Adoptive transfer therapies of T or NK cells with rapidly evolving new generations of engineered chimeric antigen receptors (CAR) are also emerging as promising alternatives to DLBCL patients. Finally, and profoundly connected with precision medicine for DLBCL patients, a plethora of small molecule inhibitors to key DLBCL deregulated pathways is continuously being investigated with the goal of inducing direct cytotoxicity of lymphoma cells. Interestingly, most of these targeted pathways are intertwined with PD-L1 regulation and might offer concomitant opportunities as immune-modulatory drugs, further offering promising clinical synergistic opportunities.

Despite the significant curative outcomes with the current standard of care R-CHOP treatment, including an anti-CD20 antibody (R, rituximab) in combination with multiagent chemotherapy (CHOP), approximately 40% of DLBCL patients experience relapse or refractory (R/R) disease [12,175]. Given the critical crosstalk between DLBCL cells and surrounding immune cells during lymphoma evolution and maintenance (Figure 2), it is reasonable to hold expectations on novel immunotherapy approaches aiming at restoring or “normalizing” [176] effective anti-tumour immunity for DLBCL. Here, we outline most promising highlights in using immunotherapy against DLBCL, with a special focus on those that directly target the PD-1/PD-L1 axis or might indirectly cooperate with this therapeutic blockade through combination (Figure 3).

### 4.1. PD-1/PD-L1 Immune-Checkpoint Blockade

Among numerous anti-PD1-/PD-L1 monoclonal antibodies (mAbs) in the global market, six currently FDA/EMA-approved mAbs to either target PD-1 (i.e., nivolumab, pembrolizumab, cemiplimab) or PD-L1 (i.e., durvalumab, atezolizumab, avelumab) are under intense investigation across multiple cancer types [177]. Despite efficacy enhancing T cell proliferation and cytotoxic functions with these antibodies in many clinical trials, PD-1 blockade with nivolumab failed to show a benefit for R/R DLBCL patients [103,178]. However, as earlier discussed, significant PD-L1 expression by the lymphoma cells, which might evidence tumour progression and more clearly associates with specific subtypes of DLBCL, may become a critical limiting factor for anti-PD-1/PD-L1 efficacy and requires further investigation as a predictive biomarker of immunotherapy responses in DLBCL. On the other hand, and contrary to de novo DLBCL, rare PD-L1 expression but high PD-1 expression are observed in the large neoplastic B cells in Richter syndrome (RS), which represents transformation of chronic lymphocytic leukemia (CLL) to DLBCL with dismal prognosis [179,180,181]. Interestingly, this distinct PD-1^hi^ phenotype in the RS lymphoma cells resembles that of protumorigenic PD-1^hi^ Breg subsets recently identified in certain cancer types [76,77], for what it has been proposed that RS patients might specially benefit from therapy targeting PD-1/PD-L1 [179,182,183,184,185,186]. Yet some discrepancies exist [187], and ongoing research is expected to shed light about the potential benefit of PD-1/PD-L1 blockade for the treatment of the DLBCL histologic variant of RS.

Finally, there is still a potential to improve the therapeutic activity of PD-1/PD-L1 blockade in B-cell malignancies and DLBCL by exploring the utility of novel combinational immunotherapy strategies, as well as by considering these therapies in a selected subset of patients with higher PD-L1 levels. Indeed, ongoing clinical trials are evaluating the efficacy of PD-1/PD-L1 blockade in combination with other agents, including antibodies targeting B-cell antigens, other suppressor or costimulatory checkpoints, bispecific antibodies to engage T cells, chimeric antigen receptor (CAR) cells, and other targeted small molecules or immunomodulatory drugs [104,174,188,189]. In this regard, preclinical data from our group support the notion that combinational immunotherapy with anti-CD20 and anti-PD-1 can reinvigorate T cells and anti-tumour specificity, improving long-term overall survival in animal models of PD-L1-positive DLBCL [133].

### 4.2. Monoclonal, Bispecific and Conjugated Antibodies

Since the approval of first-generation rituximab (anti-CD20) for the treatment of DLBCL, additional mAbs are being developed to target surface antigens in the lymphoma cells, either as unconjugated mAbs or conjugated to radioactive or cytotoxic drugs [190]. It is plausible to predict that some of these agents that directly target B-cell expressing markers (e.g., CD19, CD22, CD30, CD74, CD79b, and next generation of anti-CD20 mAbs) might also offer promising therapeutic opportunities in combination with the inhibition of PD-1/PD-L1 and other immune-checkpoints (e.g., CTLA-4, TIGIT, TIM-3, LAG-3) [189,190]. Failure to benefit from PD-1/PD-L1 blockade might also be due to effector T-cell exhaustion, which further inspires the development of mAbs targeting costimulatory receptors (e.g., CD27, OX40, CD40, 4-1BB/CD137) [189,191,192,193] to work as agonists that might overcome the immunosuppressive DLBCL microenvironment, stimulating effector T-cells and regulating T-cell memory responses. Innate immune-checkpoint blockade with anti-CD47 antibodies have also shown activity in combination with rituximab by enhancing phagocytosis of malignant tumour cells in heavily pretreated DLBCL patients [194].

On the other hand, instead of unique targeting of immune cells, bispecific antibodies targeting two antigens simultaneously are innovative synthetic constructions rapidly developing for the management of hematologic malignancies [195]. Frequently, these bispecific antibodies target one tumour antigen (e.g., CD19 or CD20 in B-cell lymphomas) and one immune-related molecule (e.g., CD3), aiming at redirecting T cells against tumour cells and working as cytotoxic T-cell engagers [196]. In DLBCL, several bispecific antibodies are under clinical evaluation with promising results, including a CD19:CD3 antibody [197], a CD20:CD3 antibody [198] and a next-generation 2:1 CD20:CD3 antibody with two CD20 binders and higher potency for hematologic malignancies [199,200].

Antibody-cytokine conjugated proteins represent another class of biopharmaceuticals in cancer immunotherapy [201]. Combination therapy of IL-21 with PD-1 or CTLA-4 previously showed efficacy in mouse tumour models [202], and an engineered IL-21 fused to a PD-1 antibody showed promising results by providing superior anti-tumour immunity than anti-PD-1 alone in preclinical studies [167].

### 4.3. CAR T-Cells and NK-Cells

Targeting DLBCL cells with anti-CD19 CAR T cells are rapidly emerging as a promising cellular immunotherapy in R/R DLBCL [203,204]. Tisagenlecleucel (tisa-cel) [205], axicabtagene ciloleucel (axi-cel) [206] and lisocabtagene maraleucel (liso-cel) [207] have shown potent therapeutic efficacy against CD19^+^ DLBCL cells. However, loss of CD19 expression or upregulation of PD-L1 have been associated with relapses after CD19 CAR T cell therapy [206,208]. In this regard, CAR T cells engineered to secrete PD-1/PD-L1 antagonists [209,210,211], to be deficient in PD-1 expression [212,213], to bispecifically target other B-cell markers (such as CD20 or CD22) in addition to CD19 [214,215], or to be administered in combination with immune-checkpoint inhibitors [216,217] are promising alternatives in the treatment of DLBCL. Furthermore, NK cells are demonstrating inherent properties that make them strong candidates for genetic modification in a CAR NK-cell format [218,219]. Indeed, clinical perspectives are continuously expanding with novel cell-based engineering strategies against CD19^+^ lymphoid tumours, many of which try to simultaneously coordinate checkpoint inhibition to enhance the function of adoptively transferred cells [220].

### 4.4. Small Molecules for Targeted Pathways and Immunomodulatory Drugs

Other agents targeting deregulated pathways in DLBCL have shown limited activity as monotherapy but are being investigated in various combinations guided by patient underlying lymphoma characteristics that might foster precision medicine for DLBCL [91,189,190]. As alluded to earlier in this review, most of these deregulated pathways have revealed capacity to ultimately impact PD-L1 levels within the DLBCL cell, highlighting a secondary immune-modulatory potential that might be further exploited by current immune-checkpoint modalities. For example, chronically-active BCR signaling in DLBCL might be inhibited with MALT1 paracaspase inhibitors [221,222] or with various kinase inhibitors such as ibrutinib or acalabrutinib (BTK inhibitors), which have shown promising clinical activities [223,224] and a potential to be efficiently combined with a variety of other agents to kill DLBCL cells [225]. Interestingly, BTK inhibition can indirectly downregulate PD-L1 signaling in DLBCL cells [132], and enhance the anti-tumour therapeutic effects of PD-1 blockade in mouse models of lymphoma [226], which might be proven synergistic with anti-PD-1 immunotherapy (NCT02362035) [227]. Inhibition of JAK/STAT3 or PI3k/AKT/mTOR pathways, which have shown potential therapeutic implications in DLBCL [228,229,230], may also ultimately impact PD-L1 expression in the tumour cells [132,231,232], providing additional rationale for future clinical evaluation with PD-1/PD-L1 blockade combinations. In selected DLBCL subtypes with aberrant toll-like receptor (TLR) signaling, IRAK4 inhibitors may have clinical utility, either alone or in combination with BTK or BCL2 inhibitors [233], with capacity to inhibit the expression of PD-L1 and provide novel opportunities for hematologic malignancies [234]. Indeed, apoptosis might be targeted with selective BCL-2 inhibitors such as venetoclax (ABT199) when added to R-CHOP [235], and is being further explored in combination with anti-PD-1 and anti-CD20 in R/R DLBCL (NCT03276468) [236]. Lenalidomide is an immunomodulatory drug with direct anti-tumour activity via binding to cereblon (CRBN) to inhibit downstream NF-kB signaling, and also indirect effects in the tumour microenvironment through multifaceted functions on different immune cells [237], including downregulation of PD-L1 on the surface of lymphoma and myeloma cells [238,239]. While discrepant results came from the addition of lenalidomide to R-CHOP in DLBCL [240,241], promising activity has been observed for triplet rituximab (anti-CD20), ibrutinib (BTKi) and lenalidomide [242,243] or in combination with rafasitamab (anti-CD19) for R/R DLBCL [244], reopening the door for this immunomodulatory drug to future clinical trials in combination [245], some of which currently involve PD-1/PD-L1 inhibitors [188].

Inhibition of exportin 1 (XPO-1) with selinexor can prevent nuclear export of key cargo proteins, including pro-tumour eIF4E-mRNAs complexes to be translated in the cytoplasm (e.g., mRNAs for c-Myc, Bcl2, Bcl-XL, Bcl6, survivin, and cyclin D1) or factors implicated in PD-1/PD-L1 upregulation (e.g., NFATC1, and STAT1/3), and this has shown substantial improved survival in R/R DLBCL patients [246] and promising efficacy in mouse syngeneic tumour models in combination with immune-checkpoint blockade [247,248].

From an epigenetic perspective, selected genetic subtypes of DLBCL might respond to targeting MYC activity with BET-bromodomain inhibitors, alone or in combination with BCL2 inhibition [249], and BET inhibition can repress PD-L1 expression and synergize with PD-1/PD-L1 blockade in mice bearing Myc-driven lymphomas [250]. Analogously, growing evidences support the promising opportunity of combining epigenetic modulators that have previously shown activity in DLBCL with immune-checkpoint blockade [251], including inhibitors for EZH2 histone methyltransferase [252,253,254], G9a/DNMT methyltransferase [255,256], or histone deacetylases [102,257,258].

## 5. Conclusions

The complex interplay between DLBCL cells and surrounding tumour microenvironment demonstrates lymphoma evolution towards the acquisition of immunosuppression and immunoescaping traits that may ensure tumour maintenance and progression. The PD-1/PD-L1 axis is a key player in these processes, likely inherited from the GC B cell-of-origin of DLBCL and engaged in disturbed inter-cellular signaling with tumour-infiltrating T cells. Evidence highlights the importance of a fine equilibrium in the proportion of intra-tumour T_FH_ and T_FR_ populations, as DLBCL cells deceive normal function of these cells to take advantage of helper pro-survival signals, while impairing regulatory control and suppressing cytotoxic functions. Selected cytokines may result pivotal in the orchestration of such pathogenic DLBCL microenvironment, where deprivation of IL-21 or addiction to IL-10 can support tumour survival and modulate PD-L1 levels in the lymphoma cells. Yet it urges us to better understand all these intricated cellular and molecular interactions in DLBCL, as this would improve the rationale for combinational immunotherapy with the constantly increasing portfolio of novel monoclonal, bispecific and conjugated antibodies, CAR T and NK cells, and small molecule inhibitors with immunomodulatory activities that hold the potential to bring more favorable outcomes for DLBCL patients.
